# Fetuin-A deficiency protects mice from Experimental Autoimmune Encephalomyelitis (EAE) and correlates with altered innate immune response

**DOI:** 10.1371/journal.pone.0175575

**Published:** 2017-04-07

**Authors:** Violaine K. Harris, Lena Bell, Ruth-Anne Langan, John Tuddenham, Mark Landy, Saud A. Sadiq

**Affiliations:** Tisch Multiple Sclerosis Research Center of New York, New York, New York, United States of America; Charite Universitatsmedizin Berlin, GERMANY

## Abstract

Fetuin-A is a biomarker of disease activity in multiple sclerosis. Our aim was to investigate whether Fetuin-A plays a direct role in the neuroinflammatory response in the mouse EAE model. Peak Fetuin-A expression in the CNS and in peripheral lymphoid tissue correlated with peak EAE disease activity. Fetuin-A-deficient mice showed reduced EAE severity associated with an accumulation of splenic monocyte and dendritic cell populations, increased IL-12p40, ASC1, and IL-1β expression, and an increase in T regulatory cells. The upregulation of Fetuin-A in LPS-stimulated dendritic cells and microglia further supports an intrinsic role of Fetuin-A in regulating innate immune activation during EAE.

## Introduction

Multiple sclerosis (MS) is a chronic autoimmune disease with an uncertain etiology characterized in the majority of patients by recurring episodes of immune-mediated demyelination in the CNS. Chronic demyelination over time leads to an accumulation of axonal loss, glial scarring, grey matter pathology, and progressive neurological disability affecting approximately 3 million people worldwide. A major objective at the time of initial diagnosis is to minimize future disability by arresting the disease early and effectively, thereby preventing later disease progression and disability. With many immune-targeting disease modifying treatments available, the challenge in assessing therapeutic intervention is the accuracy of the clinical parameters used to monitor disease activity, namely relapse rate, disability score, and MRI. There is a need for biomarkers that can detect disease activity that lies below the threshold of our current measurement tools.

Fetuin-A was recently described as a novel biomarker of disease activity in the cerebrospinal fluid (CSF) of patients with MS [1]. CSF levels of Fetuin-A were significantly elevated in MS patients with active disease as defined by a recent relapse, change in disability score, or a change in MRI outcomes. Furthermore, in MS patients treated with natalizumab, there was a significant decrease in CSF Fetuin-A levels over 6 and 12 months, compared with baseline pretreatment levels. The decline in CSF Fetuin-A levels was even more pronounced in treatment responders, compared with non-responders who showed no significant change in Fetuin-A [1]. This initial study suggested that CSF Fetuin-A could be used as laboratory measure of disease activity and treatment efficacy in MS.

Fetuin-A, a glycoprotein named based its abundance in fetal serum [2], is expressed during fetal development in certain tissues including the CNS and bone marrow [3]. In the adult, Fetuin-A is expressed predominantly in the liver [3]. In MS, Fetuin-A expression was found to be upregulated in demyelinated lesions, as well as in certain cortical and Purkinje cell neurons in MS brains [1]. Similarly in mice, Fetuin-A was upregulated in the CNS of mice with EAE, an animal model of MS [1]. Furthermore, mice lacking Fetuin-A (FAKO) had a less severe course of EAE, indicating that Fetuin-A may play a direct role in EAE pathogenesis [1].

The mechanism by which Fetuin-A may influence EAE or MS pathogenesis remains unknown. Fetuin-A is a multifunctional protein shown to have both anti-inflammatory and pro-inflammatory effects [3]. Fetuin-A belongs to the cystatin superfamily of cysteine protease inhibitors, which has been shown to inhibit transforming growth factor beta (TGF-β), hepatocyte growth factor (HGF), and matrix metalloproteinases, and is a ligand for toll-like receptor (TLR)4 [4–7]. More recently, Fetuin-A in adipocytes was associated with macrophage chemoattraction and polarization to a pro-inflammatory M1 subtype [8]. The objective of this study was to investigate how Fetuin-A may influence the immune response during EAE.

## Materials and methods

### EAE induction and adoptive transfer

All animal experiments were approved by the St. Luke’s Roosevelt Hospital Center IACUC. Heterozygous fetuin-A knockout mice have been described previously [1]. Wild-type C57BL/6J mice were purchased from Jackson Laboratory (Bar Harbor, ME). EAE induction with MOG35-55 peptide and 0–13 EAE scale have been described previously [9]. The adoptive transfer EAE model in C57Bl/6J mice was performed as previously described [10]. Briefly, EAE mice were sacrificed at day 11 and splenocytes were restimulated in culture in RPMI media with 10% FBS, 1% sodium pyruvate, 1% non-essential amino acids, Penicillin/Streptomycin, 20 μg/ml MOG 35–55 peptide (Anaspec), along with 20 ng/ml IL-12 (R&D Systems) and 10 μg/mL anti-IFN-γ (BD Pharmingen) to promote Th17 cell expansion. After 3 days, restimulated splenocytes were washed with PBS and the same number of viable cells (10–30 million cells/mouse) were injected i.p. into naïve recipient mice. Splenocytes were also analyzed by flow cytometry or for gene expression as described below. Levels of IL-17 in the supernatants were measured by ELISA (R&D Systems).

### RNA analysis

For gene expression analysis, mice were perfused with PBS and spleens and spinal cords removed and stored in RNAlater (Qiagen). For time course experiments, spinal cords were cut longitudinally and half of the tissue stored in RNAlater. Total RNA was isolated using RNeasy Lipid Tissue Mini Kit (for spinal cord) or RNeasy Mini Kit (for spleen, splenocytes, and lymph nodes) (Qiagen) and reverse transcribed using Superscript VILO Master Mix (Life Technologies). Quantitative real time PCR (qRT-PCR) was performed using TaqMan Universal PCR Mater Mix with prevalidated TaqMan gene expression assays (Life Technologies) to detect ASC (Pycard) (Mm00445747_g1), CCL2 (Mm00441242_m1), CCL5 (Mm01302427_m1), CCL7 (Mm00443113_m1), CCL19 (Mm00839967_g1), CCR4 (Mm01963217_u1), CCR7 (Mm01301785_m1), FoxP3 (Mm00475164_m1), Fetuin-A (Mm00442674_m1), Gata3 (Mm00484683_m1), H2Eb1 (Mm00439221_m1), ICAM1 (Mm00516023_m1), IFNγ (Mm00801778_m1), IL-1β (Mm00434228_m1), IL-10 (Mm00439614_m1), IL-12a (Mm00434169_m1), IL-12b (Mm00434174_m1), IL-17 (Mm00439618_m1), IL-23a (Mm00518984_m1), LTα (Mm00440228_gH), MBP (Mm01266402_m1), NFκB (Mm00476361_m1), RORc (Mm01261022_m1), Tbx21 (Mm00450960_m1), TGFβ (Mm00441724_m1), TLR4 (Mm00445273_m1), TNFα (Mm00443260_m1). Hprt1 (Mm00446968_m1) was used as an endogenous control. PCR was performed using 7900HT Fast Real-Time PCR System and results analyzed using RQ Manager software (Life Technologies). Lymph node qPCR was performed using TaqMan^®^ Array Mouse Immune Response 96-well Plate (Life Technologies) and fold changes in gene expression were determined by RT^2^ Profiler PCR Array Data Analysis software (Qiagen).

### Protein analysis by western blot

For time course analysis of protein expression, the other half of the spinal cord tissue was snap frozen. Tissue was lysed in RIPA buffer and protein concentration determined by BCA protein assay (Pierce). Proteins were separated using NuPAGE^®^ Bis-Tris polyacrylamide gels (Life Technologies) and Western blot performed with 50 ng/ml goat anti-mouse Fetuin-A antibody (R&D Systems) or anti-β-actin (Sigma), followed by HRP conjugated secondary antibody. Bands were visualized by chemiluminescence, and bands were quantitated by densitometry and normalized to β-actin. Band intensities for each time point were normalized to day 0 which was set to a value of 1.

### Immunofluorescence of EAE spinal cord tissue

EAE mice were perfused with 4% paraformaldehyde on days post-immunization (DPI) as indicated in the legend. Brain and spinal cord were removed and paraffin embedded. Transverse spinal cord sections were analyzed by immunofluorescence using anti-mouse-Fetuin-A (R&D Systems), anti-CD3 (Abcam), anti-MBP (Covance), anti-Iba-1 (WAKO), followed by Alexa-488, Alexa-555, or Alexa-647-conjugated secondary antibodies (Life Technologies). Nuclei were visualized using 4’,6-diamidino-2-phenylindole (DAPI). Fluorescence imaging was performed by confocal microscopy (Zeiss LSM 510).

Quantitation of lesion number and immune cell infiltration was performed by a blinded observer on eight individual transverse cross-sections of spinal cord per animal spanning the length of the spinal cord. Lesions were identified as areas of demyelination (reduced MBP staining) in each individual spinal cord section and counted. For CD3 and Iba-1 quantification, the number of cells positive for each marker were counted manually for all lesions and expressed relative to the tissue area.

### Flow cytometry

Mice were perfused with PBS and spinal cords were removed and dissociated using Neural Tissue Dissociation Kit (Miltenyi). Single cells were washed and centrifuged at 300 x g for 10 minutes. For myelin removal, cells were resuspended in cold 0.9 M sucrose in HBSS and centrifuged at 850 x g for 10 minutes. Supernatant containing myelin was removed by aspiration, and cells were washed in PBS and centrifuged for 3 minutes at 850 x g. To harvest spleens, mice were sacrificed by cervical dislocation, and spleens removed, dissociated using gentleMACS dissociator (Miltenyi) and strained through a 40 μm cell strainer. Red blood cells were lysed using ACK buffer (Life Technologies) and cells washed twice with PBS. Cells were resuspended in 2%FBS in PBS and Fc block (BD Pharmingen) and stained with CD45-PE-Cy7 (30 F11), CD4-FITC (RM4-5), CD8-APC (53–6.7), CD14-FITC (Sa2-8), CD11b-APC (M1/70), CD11c-APC (N418), MHC-II-PE-Cy7 (M5/114.15.2), CD80-FITC (16-10A1) (all from eBiosciences), and CD19-PE-Cy7 (1D3), Ly6C-FITC (AL-21) (all from BD Pharmingen) antibodies as described in figure legends. FITC, APC and PE-Cy7-conjugated isotypes were used as controls for gating. Mouse Regulatory T cell staining kit (ebiosciences) was used to analyze CD4^+^/CD25^+^/FoxP3^+^ cells. Analysis was performed on a FACSAria flow cytometer (BD Biosciences).

### In vitro analysis of CD11c^+^ and CD11b^+^ cells

Spleens from WT EAE mice were dissociated and CD11c+ and CD11b+ cells were isolated sequentially by positive selection using magnetic cell sorting following manufacturer’s instructions (Miltenyi). CD11c^+^ dendritic cells were isolated from mouse bone marrow and cultured as described previously [11], followed by stimulation with LPS (100ng/ml) for 48 hours. Cells were confirmed to be >90% CD11c^+^ by flow cytometry. CD11b+ primary microglia cells were isolated from brain tissue of 5-day old pups. Brain tissue was dissociated using Neural Tissue Dissociation Kit (Miltenyi) and CD11b+ cells were positively selected using magnetic cell sorting (Miltenyi). One day after plating, cells were stimulated with LPS as above for 4 or 48 hours.

### Statistical analysis

Differences between daily EAE scores, average area under the curve (AUC), and daily weights in WT and FAKO groups were analyzed by Mann-Whitney test. Data with multiple groups were analyzed by one-way ANOVA followed by Tukey’s multiple comparison post test. Data with two groups were analyzed using unpaired, two-tailed, Student’s *t*-test. Statistical significance was set to *p* values <0.05. GraphPad Prism 5 was used to calculate significance.

## Results

### Upregulated Fetuin-A in spinal cord during peak disease activity in EAE

**Fig 1:**
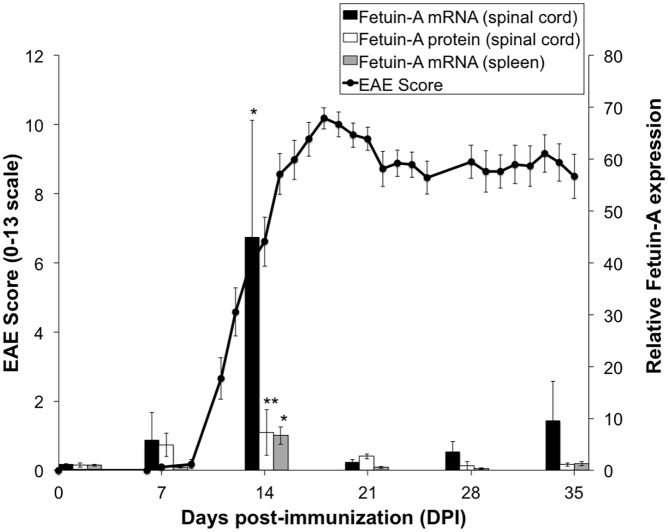
Upregulation of Fetuin-A in spinal cord and spleen during EAE. Mice with EAE were scored to assess disease severity (left axis). Mice were sacrificed each week for analysis of Fetuin-A expression in spinal cord and in spleen. Levels of Fetuin-A mRNA and protein were determined by qRT-PCR and western blot, respectively, and expressed relative to day 0 (right axis). DPI, days post immunization. EAE scores are representative of two independent experiments. Fetuin-A expression data show average of 2 independent experiments (n = 5 mice per time point per experiment). Significance shown compared to levels on day 0. *, p<0.05; **, p<0.01.

Our previous study suggested a role for Fetuin-A during EAE onset and severity, which was associated with Fetuin-A immunostaining in degenerating neurons around demyelinated spinal cord lesions in mice during late stage EAE (day 32 post-immunization) [1]. To better understand the regulation of Fetuin-A in EAE, we examined Fetuin-A expression at weekly time points during EAE. Fetuin-A mRNA was undetectable in the CNS of naïve mice and increased over the first 2 weeks of the disease with significant upregulation on day 14, correlating with rapid neurological deterioration (Fig 1). Fetuin-A levels were reduced but detectable during the later chronic phase of EAE (days 21 through 35) (Fig 1). Fetuin-A protein levels detected by Western blot followed a similar time course (Fig 1). Peak Fetuin-A expression in the spinal cord on day 14 post-immunization correlates with extensive immune cell infiltration from the periphery into the CNS [12]. We then explored whether Fetuin-A was also expressed in peripheral lymphoid tissue during EAE, and found Fetuin-A mRNA was also upregulated in EAE spleens (Fig 1) and lymph nodes (data not shown) on day 14 (Fig 1). Fetuin-A upregulation both in spleen and in the CNS coincides with peak disease activity in EAE, consistent with previous data showing Fetuin-A is a CSF biomarker of disease activity in people with MS [1]. Because Fetuin-A levels peaked on day 14, this time-point was used for the remainder of the study.

### Fetuin-A contributes to EAE severity associated with increased inflammation/demyelination

**Fig 2:**
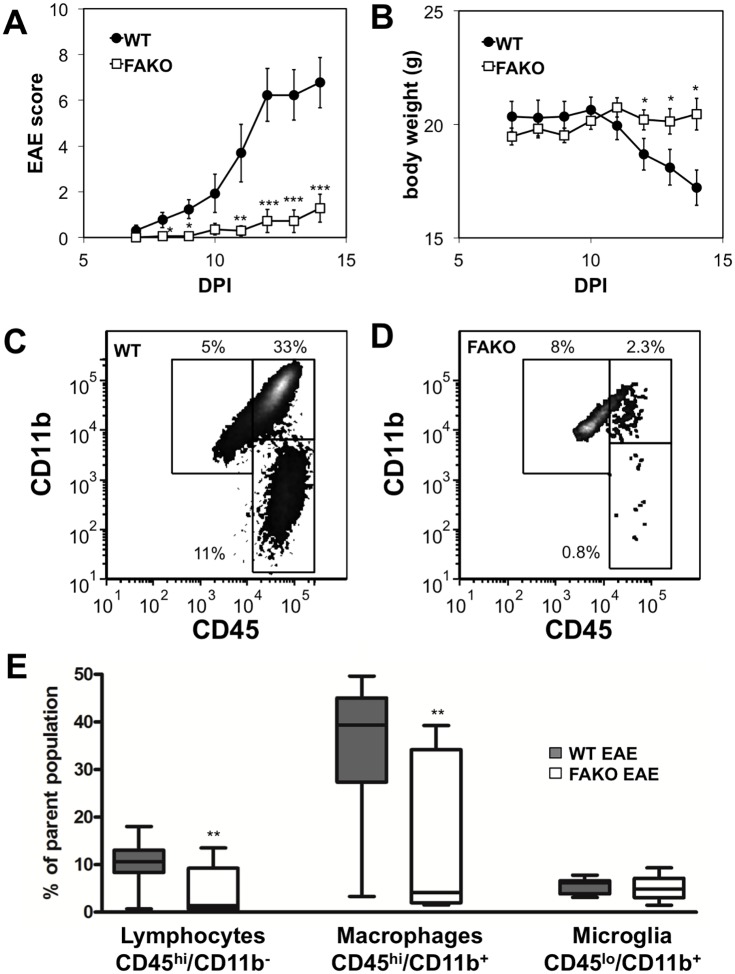
Reduced EAE severity in Fetuin-A-deficient mice associated with reduced CNS inflammation. **(A, B)** Daily average EAE scores (A), and weights (B), in wild-type (WT) C57Bl/6 mice (n = 13) or in fetuin-A homozygous knockout mice (FAKO) (n = 14). EAE severity was significantly reduced in FAKO mice. Values represent mean ± standard error and are representative of three separate experiments. Significant differences in average daily scores and weights determined by Mann-Whitney test are shown. Average AUC between the two groups was also significant (p<0.001). **(C, D)** Representative flow cytometry plots of spinal cord cells positive for CD45 and CD11b from WT (C) and FAKO (D) mice with EAE on day 14. **(E)** Quantitation of spinal cord cell populations shown in (C, D). Box and whiskers plots show significantly reduced lymphocyte (CD45^hi^/CD11b^-^) and macrophage (CD45^hi^/CD11b^+^) cell infiltration in FAKO (n = 10) compared to WT (n = 10) EAE mice. *, p<0.05; **, p<0.01; ***, p<0.001.

To further evaluate the contribution of Fetuin-A in EAE development, we utilized mice lacking Fetuin-A (Fetuin-A knockout, or FAKO), which we previously reported had delayed EAE onset, reduced disease severity, and reduced degree of spinal cord demyelination examined on day 32 [1]. Because peak Fetuin-A expression occurs 14 days after MOG immunization, we examined the differences between wild-type (WT) and FAKO EAE at this time point of peak CNS inflammation. Significantly reduced EAE severity in FAKO mice (Fig 2A and 2B) was associated with a significant reduction in lymphocyte (CD45^hi^/CD11b^-^) and macrophage (CD45^hi^/CD11b^+^) infiltration compared to WT EAE mice (Fig 2C, 2D and 2E). The percentage of microglia (CD45^lo^/CD11b^+^) was not significantly different between the two groups of mice.

**Fig 3:**
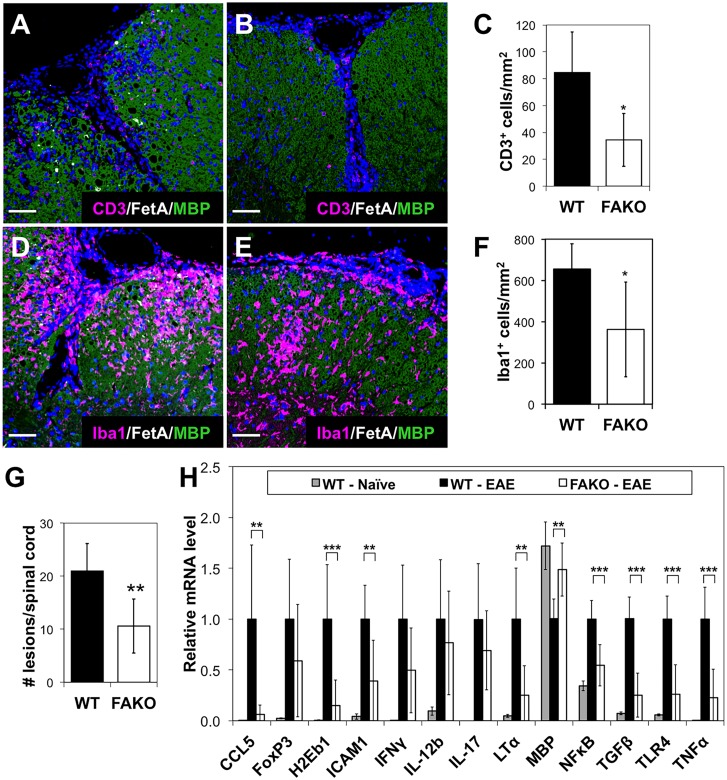
Reduced demyelination and macrophage-driven inflammation in spinal cords of EAE mice lacking fetuin-A. **(A, B)** Representative T cell infiltration in spinal cord of (A) WT and (B) FAKO EAE mice at day 14 as shown by immunofluorescence for CD3 (magenta). Demyelinated area shown by reduced MBP staining (green). Fetuin-A immunostaining shown in white, and nuclei stained with DAPI (blue). Scale bars equivalent to 50 μm. **(C)** Quantitation of CD3 immunofluorescence shown in A and B showing significant reduction of T cell infiltration in FAKO (n = 5) compared to WT (n = 5) EAE mice. **(D, E)** Representative macrophage infiltration in spinal cord of (D) WT and (E) FAKO EAE mice at day 14 as shown by immunofluorescence for Iba1 (magenta), MBP (green), Fetuin-A (white), and DAPI (blue). **(F)** Quantitation of Iba1 immunofluorescence shown in D and E showing significant reduction in macrophage infiltration in FAKO (n = 5) compared to WT (n = 5) EAE mice. **(G)** Total lesion number in spinal cords of WT (n = 5) and FAKO (n = 5) EAE mice at day 14. **(H)** mRNA expression in spinal cords from WT naïve (n = 5), WT EAE (n = 8) and FAKO EAE (n = 8) mice on day 14. Relative mRNA levels were normalized to WT EAE (value of 1). Expression levels of chemokine ligand 5 (CCL5), H-2 class II histocompatibility antigen E-B beta chain (H2Eb1), intercellular adhesion molecule 1 (ICAM1), lymphotoxin α (LT-α), nuclear factor κβ (NF-κβ), transforming growth factor β (TGF-β), toll-like receptor 4 (TLR4), and tumor necrosis factor (TNF-α) were all significantly reduced in FAKO EAE spinal cords compared to WT EAE. MBP expression was significantly higher. Values represent mean ± standard deviation. *, p<0.05; **, p<0.01; ***, p<0.001.

Immunofluorescent staining of EAE spinal cords also showed reduced CD3 (T cell) and Iba-1 (macrophage/microglia) immunoreactivity that appeared to be confined to the meninges and perivascular spaces (Fig 3B and 3D) compared to the parenchymal infiltration seen in WT EAE mice (Fig 3A and 3C). Quantitation of immunofluorescence revealed a significant reduction in T cells (Fig 3E) and macrophage/microglia (Fig 3F) in FAKO compared to WT EAE mice, and these differences correlated with a reduced number of demyelinated lesions (Fig 3G) and increased MBP gene expression (Fig 3H). These results demonstrate that Fetuin-A enhances neuroinflammation, demyelination, and neurological disability associated with the EAE model.

The immunological gene expression profile in the CNS of WT and FAKO EAE mice revealed that genes associated with innate immune response and antigen presentation, such as TGFβ, LTα, TNFα, NFκB, TLR4, and H2-Eb1 were significantly reduced in FAKO EAE compared to WT EAE (Fig 3H). Genes associated with the Th1 immune responses were slightly but not significantly reduced in FAKO EAE (Fig 3H). In addition, the EAE-associated increase in CCL5 and ICAM, both involved in leukocyte trafficking, was also reduced in the FAKO mice (Fig 3H). The reduced innate immune gene signature, along with the significant reduction in infiltrating monocytes at day 14 (Fig 2E) suggests that Fetuin-A may contribute to the peripheral immune response and prevention of macrophage and lymphocyte infiltration in the CNS.

### Fetuin-A is necessary for adoptive transfer of encephalogenic splenocytes in EAE

**Fig 4:**
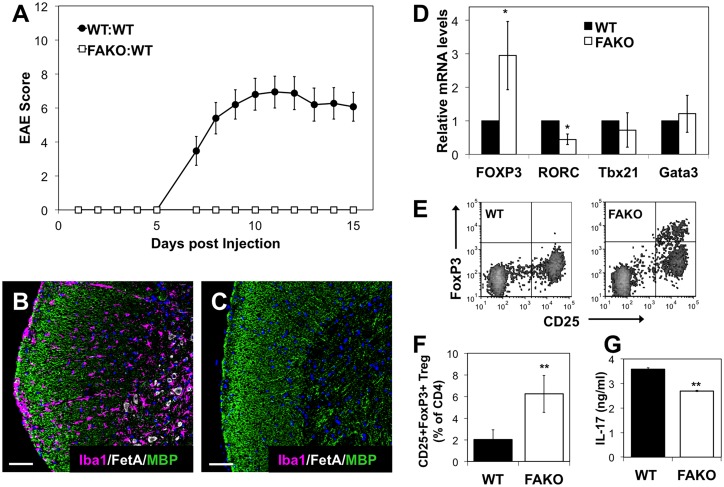
Fetuin-A-deficient EAE splenocytes do not cause EAE when adoptively transferred. **(A)** Daily average EAE scores of mice after adoptive transfer EAE. WT EAE or FAKO EAE donor splenocytes were restimulated in vitro and transferred into WT recipient mice (WT:WT) or (FAKO:WT), respectively. N = 15 mice per group. Values represent mean ± standard error and are representative of three separate experiments. **(B, C)** Representative macrophage infiltration in spinal cord of (B) WT:WT and (C) FAKO:WT EAE mice at day 14 as shown by immunofluorescence for Iba1 (magenta). Demyelinated area shown by reduced MBP staining (green). Fetuin-A immunostaining shown in white, and nuclei stained with DAPI (blue). Scale bars equivalent to 50 μm. **(D)** mRNA expression in restimulated splenocytes from WT or FAKO donor EAE mice. Relative mRNA levels were normalized to WT EAE splenocytes (value of 1). Expression levels of FoxP3 (T regulatory cells), RORC (Th17 cells), Tbx21 (Th1 cells) and Gata3 (Th2 cells) were analyzed by qRT-PCR. Values represent mean ± standard deviation compiled from three separate experiments. **(E)** Representative flow cytometry plots of CD4^+^/CD25^+^/FoxP3^+^ T regulatory cells in restimulated splenocytes from WT and FAKO EAE donor mice. **(F)** Quantitation of T regulatory cell populations shown in (E). Values represent mean ± standard deviation from a representative experiment, n = 5 mice per group. **(G)** Levels of secreted IL-17 measured from supernatants of restimulated splenocytes from WT or FAKO EAE donors. Values represent mean ± standard deviation from a representative experiment. *, p<0.05; **, p<0.01.

The expression of Fetuin-A in both the spleen and CNS during classical EAE suggests that Fetuin-A may play a dual role in peripheral immune response and in ongoing neuroinflammation. To address the contribution of Fetuin-A to peripheral immune activation in EAE, we utilized the Th17-driven adoptive transfer model of EAE whereby splenocytes from EAE mice (WT or FAKO) are restimulated with MOG peptide, IL-12, and anti-IFN-γ antibody in order to expand Th17 cells while preventing Th1 [10]. Injection of restimulated splenocytes from FAKO EAE mice into naïve WT mice was designed to examine whether a lack of Fetuin-A affects the transfer of disease in a WT CNS background. Restimulated splenocytes from WT donors resulted in transfer of EAE symptoms into WT recipients (WT:WT) approximately one week after injection (Fig 4A), which correlated with demyelination and macrophage infiltration in the spinal cords of the recipient mice (Fig 4B). In contrast, restimulated splenocytes from FAKO donors were unable to transfer EAE into WT recipients (FAKO:WT) (Fig 4A), with no apparent spinal cord pathology (Fig 4C), demonstrating that FAKO EAE splenocytes are not encephalogenic.

Restimulated splenocytes from FAKO EAE donors exhibited significantly lower expression of Th17-specific transcription factor RORc compared to WT (Fig 4D), as well as reduced level of secreted IL-17 (Fig 4G). Furthermore, FAKO donor splenocytes showed a marked increase in FoxP3 expression (Fig 4D) as well as a significant increase in percentage of CD4^+^/CD25^+^/FoxP3^+^ regulatory T cells (Fig 4E and 4F) compared to WT donor cells. Fetuin-A deficiency had no effect on cell proliferation after stimulation when analyzed by viable cell count (data not shown) or CFSE staining (S1 Fig). In addition, we found no evidence that Fetuin-A directly influences Th17 or T regulatory cell development from naïve T cells (S1 Fig). These results suggest that a lack of Fetuin-A may inhibit the peripheral autoimmune response upstream of Th17 activation in EAE, leading to compromised Th17 expansion and promotion of a T regulatory cell phenotype upon restimulation, and resulting in a lack of encephalogenicity when transferred to a WT recipient.

### Lack of Fetuin-A associated with increase of monocytes and dendritic cells in spleen

**Fig 5:**
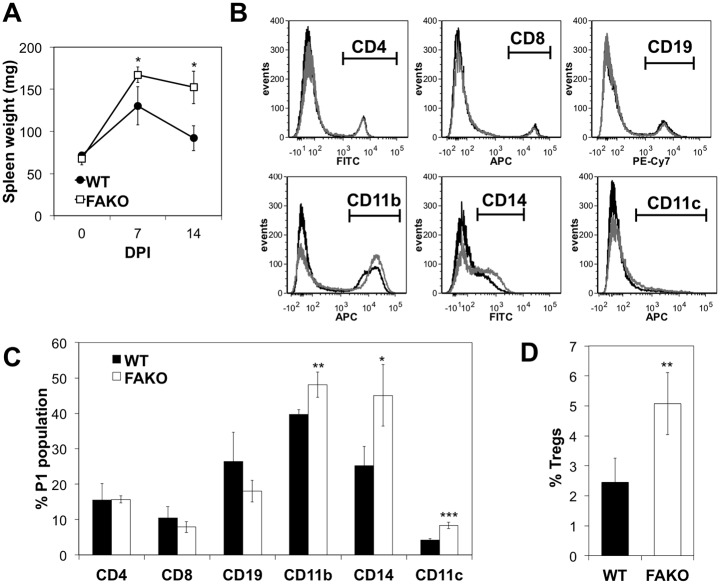
Increased splenic monocyte, dendritic cell, and T regulatory populations in EAE mice lacking Fetuin-A. **(A)** Quantitation of spleen weights in WT and FAKO during EAE on 0, 7, and 14 days post-immunization (DPI). N = 5 mice per group per time point. **(B)** Representative flow cytometry histograms of splenocytes from WT (black histogram) and FAKO (grey histogram) EAE mice on day 14. Cells were gated for CD4^+^ or CD8^+^ T-cells, CD19^+^ B-cells, CD11b^+^ or CD14^+^ monocytes, and CD11c^+^ dendritic cells. **(C)** Quantitation of spleen cell populations shown in (B) compiled from 3 separate experiments. **(D)** Quantitation of CD4^+^/CD25^+^/FoxP3^+^ T regulatory cells in spleens form WT (n = 4) and FAKO (n = 4) EAE mice, shown as percentage of gated CD4 cells. Values represent mean ± standard deviation. *, p<0.05; **, p<0.01; ***, p<0.001.

Despite their reduced disease severity, FAKO EAE mice exhibited enlarged spleen (Fig 5A) and lymph nodes (data not shown) compared to WT EAE mice. Spleen weight measured over time showed that there was a pronounced splenomegaly on days 7 and 14 in FAKO EAE mice, which was significantly higher than the transient increase in spleen weight seen during EAE in WT mice (Fig 5A). Spleen weights were identical at baseline (Fig 5A). Differences in spleen weights did not correlate with differences in body weight (Fig 2B) which appeared later in disease course and which were likely due to appetite loss at disease onset [13].

We next determined which specific immune cell populations were contributing to the increased cellularity in FAKO EAE spleens. Flow cytometry analysis of the composition of T cells, B-cells, monocytes and dendritic cells within the EAE spleens revealed that the percentages of CD4^+^ and CD8^+^ T-cells, and CD19^+^ B-cells were the same in both WT and FAKO EAE spleens (Fig 5B and 5C). In contrast, percentages of CD11b^+^ and CD14^+^ monocytes/macrophages, and CD11c^+^ dendritic cells were significantly increased in FAKO EAE spleens compared to WT (Fig 5B and 5C). Thus, the splenomegaly in FAKO EAE spleens corresponds with an accumulation of monocytes/macrophages and dendritic cells.

Although the percentages of CD4 and CD8 positive T cells were the same in both groups, we examined whether there were differences in T cell subpopulations. Consistent with the increase in T regulatory cells upon restimulation of FAKO EAE splenocytes for adoptive transfer (Fig 4E and 4F), we found a small but significant increase in the percentage of CD4^+^/CD25^+^/FoxP3^+^ T regulatory cells in FAKO EAE spleens (Fig 5D), which may contribute to the reduced disease severity in FAKO mice but does not seem to account for the increased cellularity in spleens during EAE.

**Fig 6:**
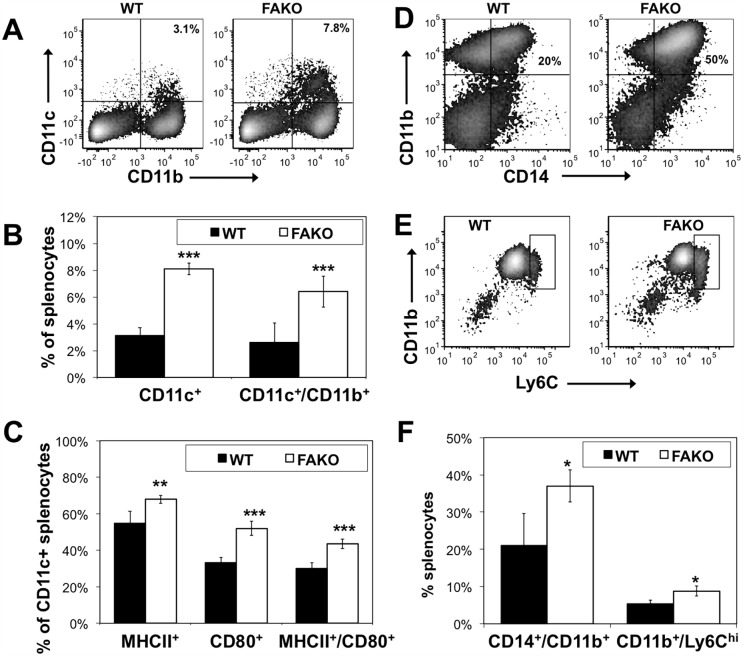
Dendritic and monocyte cell populations are increased in spleens from EAE mice lacking Fetuin-A. **(A)** Representative flow cytometry plots of dendritic cells positive for CD11c and CD11b from spleens of WT and FAKO EAE mice on day 14. **(B)** Quantitation of dendritic cell populations shown in (A). **(C)** Percentages of CD11c^+^ dendritic cells positive for CD80 and MHCII from WT and FAKO EAE mice on day 14. **(D)** Representative flow cytometry plots of CD11b^+^/CD14^+^ monocytes in spleens of WT and FAKO EAE mice on day 14. **(E)** Representative flow cytometry plots of CD14^+^ monocytes positive for CD11b and Ly6C. CD11b^+^/Ly6C^hi^ population is gated. **(F)** Quantitation of monocyte populations shown in (E) and (F). Values represent mean ± standard deviation from a representative experiment, n = 4 mice per group. *, p<0.05; **, p<0.01; ***, p<0.001.

We further analyzed splenic CD11c^+^ and CD11b^+^ cell populations in order to better understand how an increase in dendritic cell and monocyte/macrophage populations might correlate with reduced disease severity. Compared to WT EAE, FAKO EAE spleens exhibited a significant increase in double positive CD11c^+^/CD11b^+^ cells (Fig 6A and 6B), which was described previously as a tolerogenic dendritic cell population [14]. In addition, a higher percentage of CD11c^+^ dendritic cells from FAKO EAE mice were MHC-II and CD80 positive (Fig 6C). FAKO EAE spleens also showed a significant increase in CD14^+^/CD11b^+^/Ly6C^hi^ cells in compared to WT spleens (Fig 6D, 6E and 6F), corresponding to myeloid-derived suppressor cells, an anti-inflammatory population of CD14^+^/CD11b^+^ monocytes that co-express high levels of the marker Ly6C [15]. These findings show that in the absence of Fetuin-A, an increase in specific populations of CD11c^+^ and CD11b^+^ cells are associated with a diminished immune response in EAE.

### Upregulation of IL-12p40 in FAKO EAE

**Fig 7:**
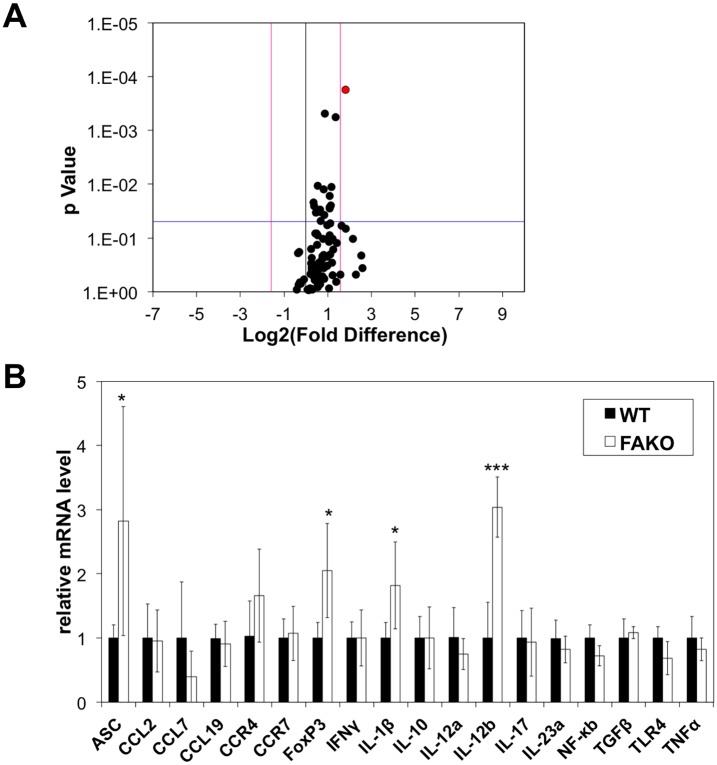
Upregulation of IL-12p40 in FAKO EAE spleens. **(A)** Volcano plot representing fold changes (log2) in gene expression in lymph nodes from WT (n = 8) and FAKO (n = 8) EAE mice on day 14. Threshold fold difference was set to 3 (log2 value 1.59) and p value was set to 0.05. Data point for IL-12p40 shown in red. **(B)** mRNA expression in spleens from WT (n = 5) and FAKO (n = 8) mice from an independent experiment on day 14. Relative mRNA levels were normalized to WT EAE (value of 1). Expression levels of ASC1, FoxP3, IL-1β and IL-12p40 were significantly elevated in FAKO EAE spleen tissue compared to WT EAE. Values represent mean ± standard deviation. *, p<0.05; ***, p<0.001.

To better understand the altered immune response in FAKO EAE, WT and FAKO EAE lymph nodes were screened for gene expression changes by PCR array containing 92 immune response associated genes. The enlarged lymph nodes in FAKO EAE mice correlated with a general increase in gene expression of immune response genes, with a significant upregulation of IL-12p40 mRNA (IL-12b) (Fig 7A). Validation of gene expression changes in spleen demonstrated that IL-12p40 mRNA was also significantly increased in spleens of FAKO EAE mice (Fig 7B). In contrast, gene expression of the other IL-12p40-interacting subunits IL-12p35 (IL-12a) and IL-23p19 (IL-23a) was unchanged, suggesting specific over-representation of IL-12p40. Additional upregulated genes included ASC1 and IL-1β, which are associated with dendritic cell inflammasome activity. Finally, FAKO EAE spleens showed increased expression of FoxP3 (Fig 7B), consistent with the increased percentage of T regulatory cells (Fig 5D). In contrast there were no changes in genes associated with Th1 (IFNγ) or Th17 (IL-17) pro-inflammatory T cell subtypes. Furthermore, there were no differences in expression levels of chemokines (CCL2, CCL7, CCL19) or chemokine receptors (CCR4, CCR7) (Fig 7B), suggesting that splenomegaly in FAKO mice is not due to defective macrophage migration [8, 16]. Although Fetuin-A was previously reported to contribute to macrophage polarization [8], there were no differences in expression of M1 (TNFα, iNOS) or M2 (IL-10, Arg1) markers in FAKO mice (Fig 7B and data not shown). The overall upregulation of pro-inflammatory molecules normally associated with activated innate immune cells suggests that the absence of Fetuin-A causes dysregulation of innate immune cell antigen presentation and/or activation necessary for development of EAE.

### Fetuin-A expression in stimulated CD11c^+^ and CD11b^+^ cells

**Fig 8:**
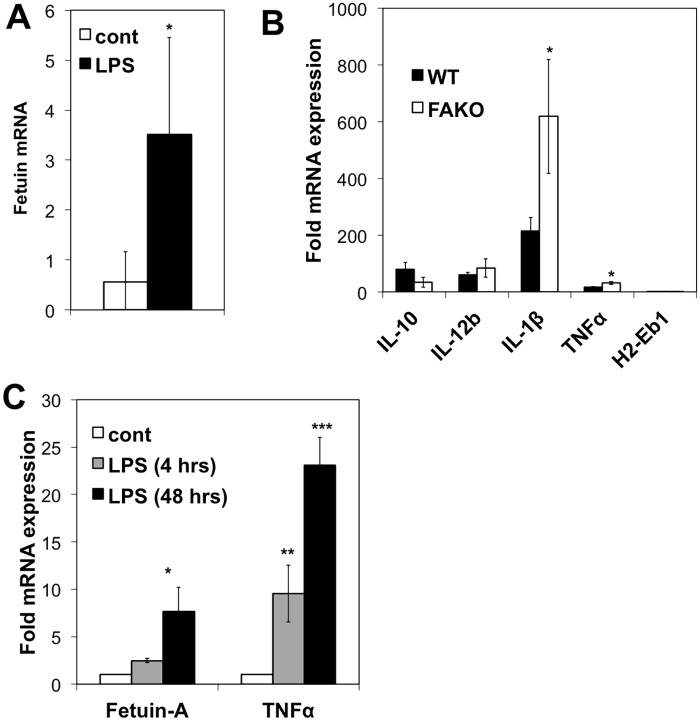
Fetuin-A expression after LPS-stimulation. **(A)** Fetuin-A mRNA expression in bone marrow-derived dendritic cells (BMDCs) stimulated with or without 100ng/ml LPS for 48 hours. **(B)** Fold change of cytokine mRNA expression after LPS stimulation of BMDCs derived from WT or FAKO mice. **(C)** mRNA expression of Fetuin-A and TNFα in primary microglia stimulated with or without 100ng/ml LPS for 4 or 48 hours. Values represent mean ± standard deviation. Data is representative of 3 separate experiments. *, p<0.05; **, p<0.01; ***, p<0.001.

To address the role of Fetuin-A in innate immune cell activation, we first examined whether Fetuin-A was expressed in CD11c^+^ and CD11b^+^ cells. As shown in Fig 1, Fetuin-A mRNA was absent in naïve spleen and but was significantly upregulated in the spleen on day 14 of EAE. Cell sorting of EAE spleens on day 14 revealed detectable Fetuin-A mRNA in CD11b^+^ and CD11c^+^ cells (data not shown). In contrast, Fetuin-A was not detectable in CD11b^-^/CD11c^-^ flow-through cells, or in naïve, Th1-polarized, Th17-polarized, or in MOG-restimulated T-cells (data not shown). To further explore the expression of Fetuin-A in CD11c^+^ cells, we cultured CD11c^+^ bone marrow dendritic cells (BMDCs) from either WT or FAKO mice. In WT-BMDCs, Fetuin-A mRNA was detectable and significantly upregulated approximately 3.5-fold upon stimulation with LPS (Fig 8A). LPS stimulation of WT BMDCs corresponded with upregulation of cytokines, including IL-10, IL-12b, IL-1β, and TNFα (Fig 8B). Interestingly, LPS-stimulated BMDCs lacking Fetuin-A resulted in an even greater upregulation of IL-1β production (Fig 8B) consistent with upregulated IL-1β production in FAKO EAE spleens (Fig 7B). We found no differences between WT and FAKO BMDCs in terms of baseline level of cytokine production or in number of cells extracted. These results suggest that like in EAE spleens, the lack of Fetuin-A in BMDCs may result in dysregulation of the pro-inflammatory response.

Fetuin-A expression in CD11b^+^ cells was investigated using a model of primary microglia isolated from neonatal mouse brain tissue. Fetuin-A expression was low but detectable in primary microglia, and was significantly upregulated along with TNFα upon stimulation with LPS (Fig 8C). The expression of Fetuin-A in LPS-stimulated cells suggests a direct role for Fetuin-A in the innate immune response.

## Discussion

In this study, we characterized the contribution of Fetuin-A to the peripheral immune response involved in the development of EAE. Our previous study identified Fetuin-A as a CSF biomarker of active disease in multiple sclerosis, with supporting data showing that Fetuin-A-deficient mice had delayed onset and reduced severity of EAE [1]. The current study extends this observation by elucidating perturbations in innate immune cell populations that impair EAE development as a result of Fetuin-A deficiency. We found that peak expression of Fetuin-A both in spleen and in spinal cord on day 14 of EAE correlates with the acute onset of tail and limb paresis/paralysis caused by extensive immune cell infiltration from the periphery into the CNS [12]. The markedly milder clinical course of EAE in Fetuin-A-deficient mice was accompanied by diminished inflammatory infiltrate into the spinal cords of mice and less severe spinal cord pathology including fewer lesions and less demyelination. The contribution of Fetuin-A to the peripheral immune response to MOG was demonstrated using the adoptive transfer EAE model whereby splenocytes from Fetuin-A-deficient donor EAE mice were unable to transfer encephalogenicity to a wile-type host. Thus, CNS expression of Fetuin-A does not seem play a role in EAE onset, and additional research will be required to determine its role, if any, in ongoing neuroinflammation. The current study demonstrates that loss of Fetuin-A causes a shift in innate immune populations in the spleen with overrepresentation of dendritic and monocyte cell populations with a suppressive phenotype along with an increase in T regulatory cells, suggesting that Fetuin-A functions to regulate the initial innate immune response in EAE.

The pro-inflammatory role of Fetuin-A can occur through multiple mechanisms including the modulation of TGFβ [4] and TLR4 [7]. TGFβ is known to exert a protective effect in EAE by downregulation of pro-inflammatory cytokines, promotion of T regulatory cells, and prevention of sensitized T cells from entering into the CNS [17]. Conversely, TGFβ also plays role in promoting autoimmune disease through its role in the differentiation of pro-inflammatory Th17 cells [17]. Our study suggests that Fetuin-A may exert some, but not all, of its pro-inflammatory effects through TGFβ inhibition, since loss of Fetuin-A mimics TGFβ effects on the promotion of tolerogenic dendritic cell and T regulatory cell populations. Despite this correlation, the expected changes in IL-10, IL-17, or TGFβ expression were not detected in the spleens of FAKO EAE mice. Furthermore, TGFβ-mediated naïve T cell polarization experiments failed to show any direct contribution of Fetuin-A to development of Th17 or T regulatory cell populations. We also did not observe any gene expression changes in TGFβ, TNFα, or IL-10, suggesting that TGFβ-signaling in DCs is not specifically perturbed in Fetuin-A knockout mice [14].

Along with a dramatic reduction in EAE severity, the loss of Fetuin-A resulted in an accumulation of DC and monocyte populations in the spleen. The specific increase in DC and monocyte populations with immunosuppressive function [14, 15] correlated with increased percentage of T regulatory cell in the spleen, along with increased FoxP3 expression. Despite its pro-inflammatory role in EAE, Fetuin-A-deficiency caused a counter-intuitive upregulation of pro-inflammatory genes including IL-12p40, IL-1β, and ASC. IL-12b (p40 subunit) is expressed by activated antigen presenting cells including macrophages and dendritic cells and functions as the common subunit for pro-inflammatory cytokines IL-12 and IL-23 [18], where it promotes Th1 and Th17 responses, respectively. It is unclear how the upregulation of IL-12p40 might play a role in protection from EAE. We speculate that the formation of IL-12p40 homodimers that might antagonize IL-12 function and promote the recruitment of myeloid-derived suppressor cells [19, 20]. Alternatively, the upregulation of IL-12p40 subunit may reflect specific dysregulation of TGFβ or TLR4 signaling, which are known to modulate IL-12p40 expression [21, 22].

ASC is one of the three protein components of the inflammasome, which plays a critical role in the ability of innate immune cells to promote EAE autoimmunity [23]. The inflammasome functions to process IL-1β, which was also upregulated in FAKO EAE mice. Interestingly, ASC1 knockout mice also exhibit splenomegaly associated with reduced EAE severity and reduced migratory ability of effector T cells [24]. In the case of FAKO EAE, however, ASC1 is upregulated rather than downregulated, and we found no difference in the expression of other inflammasome components such as NLRP3. The exact mechanism by which Fetuin-A normally contributes to antigen presentation, activation, and/or migration of DCs remains to be determined. The increased cytokine expression in FAKO spleen and BMDCs suggests that loss of Fetuin-A exerts an intrinsic dysregulation of innate immune cells that ultimately prevents EAE development.

An intrinsic role for Fetuin-A during innate immune cell activation is further supported by the finding that Fetuin-A is expressed in CD11b^+^ and CD11c^+^ cells both during peak disease activity in EAE, and in LPS-stimulated in vitro models. Fetuin-A has been shown previously to modify the LPS response in macrophages [25], and thus the upregulation of Fetuin-A may indicate an inhibitory feedback loop to modulate LPS stimulation. Like LPS, Fetuin-A can bind TLR4 and mediate fatty acid-induced inflammation in adipose tissue by promoting macrophage migration and polarization to the pro-inflammatory M1 phenotype [7, 8]. We did not observe any differences in in expression of M1 (TNFα, iNOS) or M2 (IL-10, Arg1) markers in FAKO mice in the spleen of FAKO EAE mice, although further investigation will be required to determine any impact of Fetuin-A on macrophage polarization in the CNS during EAE.

Elevated Fetuin-A levels in CSF of patients with MS correlates with disease activity and has been shown to be a useful biomarker for clinical monitoring of treatment response to natalizumab [1]. Similarly in the mouse, Fetuin-A correlates with disease activity, where it plays a key role in the development of the peripheral innate immune activation during EAE. Overall, this study helps define the role of Fetuin-A as a biomarker of the immune response in MS.

## Supporting information

S1 Fig Fetuin-A does not directly affect T cell polarization or proliferation. **(A)** Naïve CD4+ T cells from Fetuin-A-deficient mouse splenocytes were capable of Th17 polarization when compared to WT cells. Control cells were stimulated with anti-CD3/CD28 and IL-2 only, and Th17 polarized cells were stimulated with anti-CD3/CD28, IL-2, IL-1β, IL-23, TGF-β, IL-6, anti-mouse IL-4, and anti-mouse IFN-γ. Percentage of CD4^+^/IL-17^+^ positive cells was determined by flow cytometry. **(B)** There was no difference in proliferation in WT or FAKO-derived T cells when stimulated under control or Th17-polarizing conditions. Added Fetuin-A had no effect on proliferation. Proliferation was measured by reduced mean fluorescence intensity (MFI) of CFSE-labeled cells when compared to unstimulated controls. **(C, D)** Addition of Fetuin-A does not affect Th17 cell polarization of human T cells when analyzed by expression of Th17-associated transcription factor RORc (C) or IL-17 cytokine production (D). **(E, F)** Addition of Fetuin-A does not affect Treg cell polarization of human T cells when analyzed by expression of Treg-associated transcription factor FoxP3 (E) or percentage of CD4^+^/FoxP3^+^ T cells (F).
(TIF)

S1 Methods Materials and methods for S1 Fig. (DOCX)
