# Supplementary figures and images for: Fetuin-A deficiency protects mice from Experimental Autoimmune Encephalomyelitis (EAE) and correlates with altered innate immune response
Source: PLoS One. 2017 Apr 7;12(4):e0175575. doi: 10.1371/journal.pone.0175575 (PMC5384772; doi:10.1371/journal.pone.0175575)

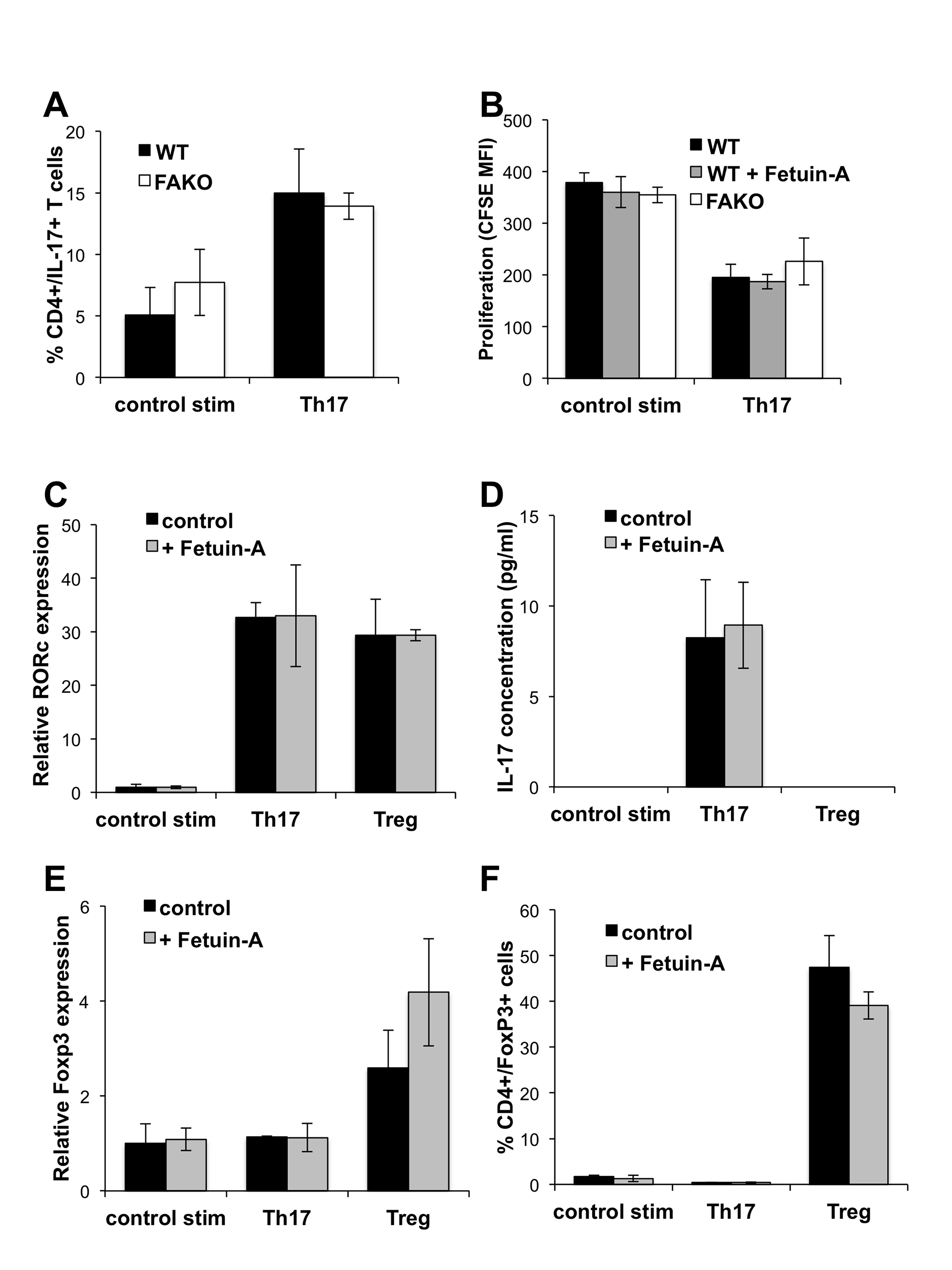

Supplement: S1 Fig — (A) Naïve CD4+ T cells from Fetuin-A-deficient mouse splenocytes were capable of Th17 polarization when compared to WT cells. Control cells were stimulated with anti-CD3/CD28 and IL-2 only, and Th17 polarized cells were stimulated with anti-CD3/CD28, IL-2, IL-1β, IL-23, TGF-β, IL-6, anti-mouse IL-4, and anti-mouse IFN-γ. Percentage of CD4+/IL-17+ positive cells was determined by flow cytometry. (B) There was no difference in proliferation in WT or FAKO-derived T cells when stimulated under control or Th17-polarizing conditions. Added Fetuin-A had no effect on proliferation. Proliferation was measured by reduced mean fluorescence intensity (MFI) of CFSE-labeled cells when compared to unstimulated controls. (C, D) Addition of Fetuin-A does not affect Th17 cell polarization of human T cells when analyzed by expression of Th17-associated transcription factor RORc (C) or IL-17 cytokine production (D). (E, F) Addition of Fetuin-A does not affect Treg cell polarization of human T cells when analyzed by expression of Treg-associated transcription factor FoxP3 (E) or percentage of CD4+/FoxP3+ T cells (F). (TIF) [file pone.0175575.s001.tif]
