# Supplementary material for: Fetuin-A deficiency protects mice from Experimental Autoimmune Encephalomyelitis (EAE) and correlates with altered innate immune response
Source: PLoS One. 2017 Apr 7;12(4):e0175575. doi: 10.1371/journal.pone.0175575 (PMC5384772; doi:10.1371/journal.pone.0175575)
Supplement: S1 Methods — (DOCX) [file pone.0175575.s002.docx]

**S1 Methods**

T cells were isolated from dissociated mouse spleens from either WT or FAKO mice using mouse naïve CD4^+^ T cell isolation kit (Miltenyi). Human T cells were purified peripheral blood mononucleated cells using CD4+ T cell isolation kit II (Miltenyi). Cell were cultured in IMDM + 5% FBS and stimulated with either mouse or human T-Activator CD3/CD28 Dynabeads® according to manufacturers instructions in the presence of 10 ng/ml mouse or human IL-2. For Th17 polarization the following mouse or human cytokines were added: 50 ng/ml IL-1β, 50 ng/ml IL-23, 5 ng/ml TGF-β, and 25 ng/ml IL-6. In addition, 1 mg/ml anti-mouse IL-4 (Clone 11B11) and 1 mg/ml anti-mouse IFN-γ (clone XMG1.2) or 1 mg/ml anti-human IL-4 (clone MP4-25D2) and 1 mg/ml anti-human IFN-γ (clone B27) were added to mouse or human cells, respectively, to block Th1 and Th2 polarization. For T regulatory cell (Treg) polarization, only 5 ng/ml TGF-β was added. All cytokines were purchased from R&D Systems and all antibodies were from BD Pharmingen. In some experiments, 120 μg/ml purified Fetuin-A (EMD Millipore) was added. Cells were harvested after 4 days.

For IL-17 staining, cells were restimulated with 50 ng/ml PMA, 1 μg/ml Ionomycin (both from Sigma), and BD GolgiStop™ (monesin) for 4 hours and were analyzed by flow cytometry as described in methods using IL-17-PE (eBio17B7, eBiosciences) and CD4-Pacific Blue (RM4-5, BD Pharmingen) or isotype controls. Human Tregs were detected by flow cytometry using regulatory T cell staining kit (eBioscience). For proliferation assays, cells were labeled with 2 μM carboxyfluorescein succinimidyl ester (CFSE) and proliferating cells were gated compared to unstimulated control. Reduced CFSE mean fluorescence intensity (MFI) was indicative of increased proliferation.

IL-17 concentration in conditioned media was determined by ELISA (R&D Systems). Gene expression was analyzed by TaqMan-based qRT-PCR as described in methods using primers for RORc (Hs01076112_m1) and Foxp3 (Hs01085832_m1). 18S (Hs99999901_s1) was used as an endogenous control.
